# Corneal Stability following Hyperopic LASIK with Advanced Laser Ablation Profiles Analyzed by a Light Propagation Study

**DOI:** 10.1155/2018/3060939

**Published:** 2018-03-28

**Authors:** Almutez M. Gharaibeh, Asier Villanueva, David Mas, Julian Espinosa, Jorge L. Alió

**Affiliations:** ^1^Department of Ophthalmology, The University of Jordan Faculty of Medicine, Amman, Jordan; ^2^Vissum Instituto Oftalmológico de Alicante, Universidad Miguel Hernández, Alicante, Spain; ^3^Department of Optics, University of Alicante, Alicante, Spain; ^4^Division of Ophthalmology, Universidad Miguel Hernández, Alicante, Spain

## Abstract

**Purpose:**

To assess anterior corneal surface stability 12 months following hyperopic LASIK correction with a light propagation algorithm.

**Setting:**

Vissum Instituto Oftalmológico de Alicante, Universidad Miguel Hernández, Alicante, Spain.

**Methods:**

This retrospective consecutive observational study includes 37 eyes of 37 patients treated with 6th-generation excimer laser platform (Schwind Amaris). Hyperopic LASIK was performed in all of them by the same surgeon (JLA) and completed 12-month follow-up. Corneal topography was analyzed with a light propagation algorithm, to assess the stability of the corneal outcomes along one year of follow-up.

**Results:**

Between three and twelve months postoperatively, an objective corneal power (OCP) regression of 0.39 D and 0.41 D was found for 6 mm and 9 mm central corneal zone, respectively. Subjective outcomes at the end of the follow-up period were as follows: 65% of eyes had spherical equivalent within ±0.50 D. 70% of eyes had an uncorrected distance visual acuity 20/20 or better. 86% of eyes had the same or better corrected distance visual acuity. In terms of stability, 0.14 D of regression was found. No statistically significant differences were found for all the study parameters evaluated at different postoperative moments over the 12-month period.

**Conclusions:**

Light propagation analysis confirms corneal surface stability following modern hyperopic LASIK with a 6th-generation excimer laser technology over a 12-month period.

## 1. Introduction

Laser in situ keratomileusis (LASIK) when used to correct hyperopia and hyperopic astigmatism accomplishes hyperopic corrections that occur through ablating an annular zone at the periphery of the cornea. The desired refractive effect is achieved by causing relative flattening of the corneal periphery and concomitantly relative steepening of the center (optical zone).

The results of post-LASIK ablation for hyperopic correction have been traditionally considered as safe [1], predictive, and effective procedure [2, 3], albeit less precise and stable in comparison to those for myopic correction [3–5]. Therefore, patient's satisfaction has been considered more limited. Decentration, large optical zone size with induction of more aberrations, peripheral ablations, latent hyperopia, and regression are some of the causes for such dissatisfaction [4].

Hyperopia regression comes from two ways principally, corneal regression, caused by the progressive change in the shape of the cornea after the LASIK intervention and the flattening of the central area causing a decrease in the corneal power; there are different theories of why this happens. The development of new generations of lasers and nomograms tries to reduce this issue. And, the latent hyperopia, detected only by cycloplegia, are caused by the tonic accommodation of the lens; this excessive tonic state of accommodation is caused by the intent of the visual system to compensate the hyperopia. In some patients, usually related with high amounts of latent hyperopia, if the total amount of cycloplegic refraction is corrected, when the effect of the cycloplegic drugs ends, the eye tries to recover values close to its “altered tonic state” of accommodation, causing a pseudomyopia and patient discomfort.

Hyperopic patients are also capable of compensating latent hyperopia which might manifest posttreatment as undercorrection or regression. This might even become manifested with age after LASIK correction [6]. When analyzing hyperopic LASIK outcomes, regression should be differentiated from decompensated latent hyperopia when evaluating decreasing efficacy post-LASIK ablation for hyperopia. Some studies based on subjective manifest refraction, post-LASIK surgery, have shown significant regression in the first 12 months after surgery with more stable results from 12 months postoperatively [7].

Light propagation algorithm analysis of the corneal surface is a purely objective method based on behavior of light through a refractive surface of the ocular media; analyzing the focal behavior of the light as affected by an optical surface or refractive media in any given diameter may help us understand corneal behavior after hyperopic LASIK treatment. This type of analysis is more comprehensive than the simple analysis of the corneal topography as it offers the refractive behavior of a chosen area of the anterior corneal surface in a global form rather than the focal one obtained when the study is performed by the analysis of the keratometry values in given meridians. For this reason, we have used for the very first time to the best of our knowledge in the analysis of hyperopia, regression following LASIK, a light propagation algorithm previously tested by our group in other studies [8]. The method calculates the corneal transmittance from height data from the first and second corneal surface and local corneal thickness. From the transmittance function, the propagated light pattern or the modulated transfer function can be calculated at any desired plane [9, 10]. Common method based on ray tracing needs impact dots statistics to obtain the final energy in a plane, thus imposing a different sampling grid at each considered axial distance. Our method avoids this problem so results are more accurate. Because of this, the method is computationally very efficient, allowing the evaluation of more than 10,000 elevation points simultaneously, thus allowing a very realistic modelling of the cornea [9, 11].

The primary objective of this study is to investigate with an objective method of wavefront propagation analysis the anterior surface corneal stability following LASIK correction in hyperopic eyes ablated with a 6th-generation excimer laser technology. To the best of our knowledge, this is the first study to address the corneal stability following corneal refractive surgery and precisely following hyperopic LASIK using wavefront propagation analysis of the anterior corneal surface.

## 2. Methods

This was a consecutive, observational case series study which comprised patients who had LASIK to correct hyperopia with a 6th-generation excimer laser device and with a postoperative follow-up of 12 months. Before surgery, each patient was adequately informed about the surgery, its risks, and its benefits. Each patient signed an informed consent in accordance with the tenets of the Declaration of Helsinki. Approval from the Ethical Board Committee of our institution was granted for this investigation to perform the retrospective analysis of this series of cases.

Inclusion criteria for hyperopic LASIK surgery were hyperopia between +0.50 and +8.50 diopters (D) and/or hyperopic astigmatism up to 4.75 D, with the presence of patient motivation for the refractive surgical correction of their refractive error. According to pachymetry, a calculated postoperative corneal stromal bed thickness of more than 250 mm at the thinnest corneal area was to be left after surgery. Contact lens wearers were advised to discontinue their use for at least 4 weeks before surgery.

Exclusion criteria were patients younger than 18 years old, unstable hyperopia, presence of active corneal disease, uveitis, anterior or posterior synechiae, glaucoma or ocular hypertension, history of ocular trauma, previous intraocular or corneal surgery, retouch of refractive surgery, irregular cornea on corneal topography, and preoperative mean keratometry outside the range of 39.00 to 46.00 D; patients with amblyopia and a potential CDVA of less than 0.4 on the decimal visual acuity scale (20/50) were also excluded.

To avoid bias of selection, only the right eye of this consecutive series was always chosen to be included in the study. All topographies used for the purpose of propagated field analysis were obtained at 1-week preop and 3 and 12 months postop using the same Placido ring-based corneal topography.

Some studies suggest that regression is strongly related with the amount of hyperopia; so to ascertain the influence of the amount of the SE in the results, patients were analyzed all together and subdivided and analyzed in group 1 (SE ≤ 3) and group 2 (SE > 3).

### 2.1. Light Pattern Analysis: Objective Calculation of the Focus of the Anterior Corneal Surface

Our method has been developed using the proprietary software MATLAB R2016b (The Mathworks, Natick, USA) which considers height data obtained from corneal topographies taken at different moments. For calculation proposes, the time delay, evaluated through the optical path, between different locations of a planar wavefront entering the cornea is considered. To do so, we have defined an input plane, tangent to the corneal apex, and an output one arbitrarily situated behind the cornea. In this study, we have not considered the second corneal surface since it contributes to a very small fraction of the total eye power. Nevertheless, its overall effect has been considered by using an equivalent keratometric index *n*_k_ = 1.3375.

Between the two planes, we calculate the optical path using the following equation:

(1) $$\begin{array}{c} L_{T}=n_{air}\times d_{IC}+n_{k}\times d_{CO}\equiv c\times t_{IO}, \end{array}$$

with *n*_air_ and *n*_k_ being the air and keratometric indexes and *d*_IC_ and *d*_CO_ being the distances between the input plane and the cornea and the cornea to the output plane at a specific coordinate; *c* and *t* are the speed of light and the time that the light would take in travelling from the input to the output plane in the vacuum at that coordinate. The distance between the input plane and the cornea is obtained from the elevation map obtained thought optical topography. In order to have better accuracy, the whole surface is fitted with 35 Zernike polynomials, and then it is analytically interpolated in order to have the elevation at any desired point through each of these points, a rectilinear trajectory is traced and the optical path for this particular trajectory is obtained.

The wavefront inside the cornea is constructed by obtaining the surface that joins all the points with the same optical path at a time. From the obtained wavefront, we can obtain the light distribution at any plane inside the eye just by using a propagation algorithm. By this, we are only assessing the optical quality of the cornea ignoring the effect of any other ocular structures (e.g., lens).

The best image plane is established by considering the energy density, that is, light intensity per subtended degree, measured from the image nodal point of the cornea. The higher the density, the best the image quality is expected. Time stability of the intervention is analyzed through the relative displacement of the maximum location.

The approach based on wavefront propagation provides accurate and reliable results not only in ophthalmic optics [12] but also in optical design [13, 14].

**Figure 1:**
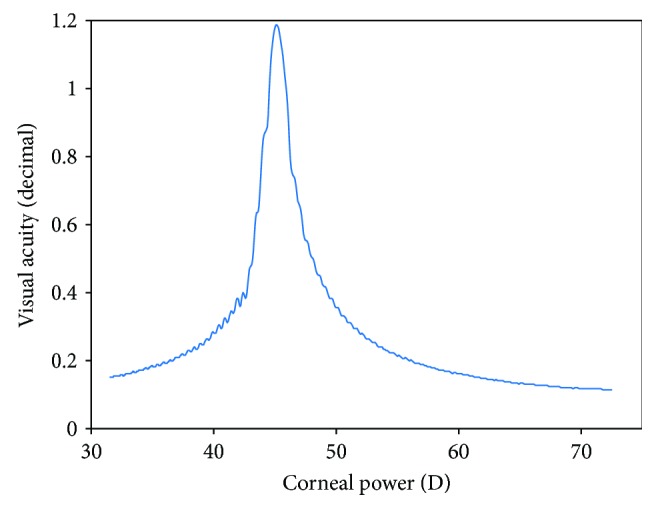
Axial visual quality versus corneal power. The peak is taken as the OCP.

**Figure 2:**
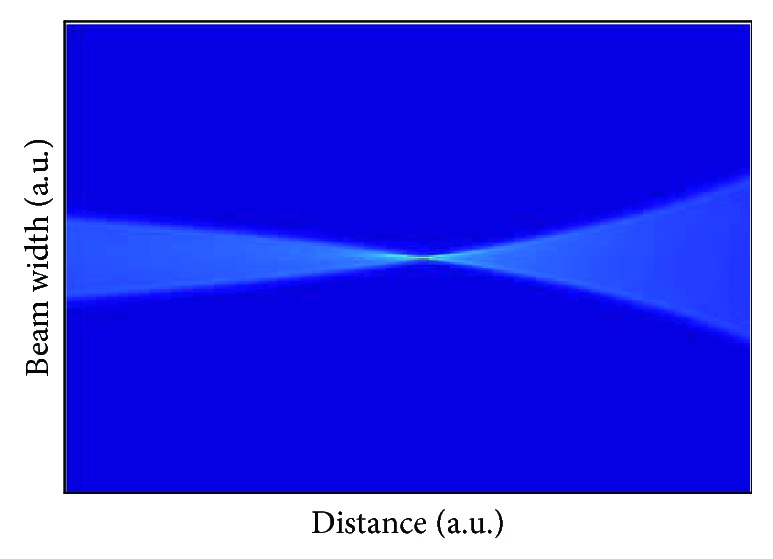
Simulation of rays inside the eye.

Computationally, the Fresnel propagation method is more efficient than those based on classical ray tracing. This permits using a denser sampling and therefore considers the entire corneal surface instead of average values obtained from keratometry, with the result being more realistic and accurate. Figures 1 and 2 represent a result window that the developed software provides and a simulation of behavior of light across the cornea of one patient, respectively. The result obtained with this method is equivalent to the ocular point spread function (PSF) at any distance inside the eye, with a vergence resolution of 0.001 D [15, 16]. The propagation algorithms can be applied in cascade starting from any arbitrary point results can be calculated sequentially, so it permits adding intermediate elements like the crystalline lens or intraocular lens (both diffractive and refractive) almost immediately. It also permits immediate calculation of the modulation transfer function (MTF) thus enabling a complete analysis of refractive status of the eye, including aberrations, PSF analysis, radial MTF, and so on.

In the cases we are dealing here, after a hyperopic LASIK treatment, spherical-like aberrations are increased [4, 5] and spherical aberration increases deep of focus (DOF) of the cornea [17, 18], so in a subjective exploration of the refraction, the protocol is set to put “the maximum positive with the best visual acuity.” In a repeated subjective refraction, the examiner can introduce a bias in the result by not putting the cutoff value of sphere on the same place of this extended DOF corneal range, because we are reacting to the patients' response, and in an extended DOF region, the answer may not be clear.

With our proposed objective analysis, the analysis is always made based on the entire behavior of the light through the analyzed ocular media; in this case, the anterior corneal surface and the focus are allocated in the middle of the extended DOF region. Following these criteria, this method is consequently more accurate than the subjective refractive analysis.

### 2.2. Excimer Laser and Ablation Profile

All LASIK procedures were performed by one experienced surgeon (JLA) at Vissum Instituto Oftalmológico de Alicante, Spain, following the same surgical protocol. A 60 kHz Intralase femtosecond laser (Intralase, Advanced Medical Optics Inc.) was used to create the flap. In all cases, a flap diameter of 9.12 mm with a depth of 110 mm was planned. LASIK was done using the sixth-generation Amaris excimer laser (Schwind eye-tech-solutions GmbH and Co., Kleinostheim, Germany) [19]. This system has a fast repetition rate (500 Hz) and incorporates two levels of fluence. A high fluence level (i.e., ablative spot profile of 0.8 mm) is used in the first 80% of the treatment to speed up the procedure. For the remaining 20% of the treatment, [20] a low fluence level (i.e., ablative spot profile of 0.72 mm) is used to ensure the smoothness of the ablated surface. A small beam (0.54 mm) with a super-Gaussian ablative spot profile is delivered in a randomized flying-spot pattern to reduce the successive overlapping of the laser spot and minimize the thermal load to the cornea [21]. All treatments performed in the current series were based on optimized aspheric ablation profiles and calculated using ORK-CAM software (Schwind eye-tech-solutions GmbH and Co.). The temperature and humidity conditions in the excimer laser operating room were continuously maintained within the ranges stated by the laser manufacturer.

The optimized aspheric aberration-free profiles consider a compensation factor for the loss of efficiency when the laser hits the cornea in a nonnormal incidence; the goal is to avoid inducing aberrations and balance the aberrations that are present in the treated eye. With the same goal, a multiaspheric transition zone is always created [22].

The ablation was performed as a peripheral ring. The optical zone of the ablation area was between 6.2 mm and 6.9 mm, with most cases having a 6.5 mm optical zone and a 1.5 mm transition zone. Like Frings et al. [23] suggest, in order to minimize the effect of latent hyperopia, the planned refraction to be corrected for patients younger than 40 years was the manifest refraction plus 50% of the difference between the manifest refraction and the cycloplegic refraction. For patients older than 40 years, the manifest refraction was to be corrected. Ablations were centered on the corneal vertex using half the pupillary offset, that is, the distance between the pupil center and the normal corneal vertex measured by videokeratoscopy. The pupillary offset measurement was translated into the treatment plan as Cartesian coordinates and then manually entered in the excimer laser computer. Alignment of the eye during the excimer laser procedure was insured via the infrared eye tracker integrated in the system, with simultaneous limbus, pupil, and torsion tracking integrated into the system and centered on the vertex of cornea.

The distance between the deepest points of both edges of the peripheral ring yields the optical zone diameter, which was a minimum of 6.2 mm in this study. This gives a transition zone (a nonoptical zone designed to blend the optically ablated zone with the untreated area of the cornea) of approximately 2.0 mm from the deepest spot to the nonablated cornea on each side, with a consequent total ablation diameter between 8.1 mm and 9.5 mm. The angle *k* must be considered before hyperopic refractive surgery [24], because hyperopic patients have a greater positive angle *k* than emmetropic and myopic patients [25]. For this reason, the hinge was temporal in all cases to avoid interfering with the angle *k* [26].

### 2.3. Preoperative and Postoperative Management

The preoperative examination included uncorrected distance visual acuity (UDVA); corrected distance visual acuity (CDVA); manifest refraction using the fogging method and cycloplegic refraction after 30 minutes of the instillation of two drops of cyclopentolate separated by 20 minutes; slit-lamp biomicroscopy; ultrasonic pachymetry (Ocuscan RxP; Alcon Laboratories Inc., Fort Worth, TX); Goldmann tonometry; scotopic, low mesopic, and high mesopic pupillometry (Procyon Pupillometer P2000SA; Procyon Instruments Ltd., Stirling, United Kingdom); corneal topography (Eyetop; CSO, Firenze, Italy); and fundus evaluation. After surgery, patients were treated with a standard combination of tobramycin and dexamethasone (Tobradex; Alcon Laboratories Inc., Fort Worth, TX) eye drops four times daily for 1 week. Postoperative examinations were at 1 day and 3, 6, and 12 months.

For statistical purposes, visual acuity was set on logMAR. An independent observer performed the examinations and collected the data.

### 2.4. Statistical Analysis

Statistical analysis was performed with the SPSS statistical software package, version 23.0 for Windows (SPSS Inc., Chicago, IL). To analyze the correlation between the three moments of interest (preoperatively, 3 months, and 1 year postoperatively), one-way ANOVA for repeated measures was used when parametric analysis could be applied. When nonparametric tests should be applied, the nonparametric Friedman test (exact test) was applied. When necessary normality was confirmed by Kolmogorov-Smirnov with Lilliefors correction, sphericity was confirmed by the Mauchly test. When sphericity is not present, Greenhouse-Geisser and Huynh-Feldt corrections were set. To study the relations between pairs of groups, ANOVA's pairwise comparisons with Bonferroni correction were used. When nonparametric test was necessary, Wilcoxon rank-sum test with Bonferroni adjustment was used. Main outcomes measured were summarized and all of the *p* values are associated with the correspondent test in (Tables 1, 2, and 3).

Differences were considered statistically significant when the associated *p* value was less than 0.05.

## 3. Results

Thirty-seven eyes of consecutive thirty-seven patients operated between January 2015 and January 2016 were fit (as per the inclusion criteria) for the study. Their preoperative cycloplegic refractive spherical equivalent (SE) ranged from +0.625 to +8.0. Demographics of the patients are shown in (Table 1). Subjective outcomes are presented in standardized figures [27].

To see the influence of the amount of the SE on the results, patients were analyzed all together (Table 1) and subdivided and analyzed in group 1 (SE ≤ 3) and group 2 (SE > 3). Results of group 1 and group 2 are summarized in (Tables 2 and 3), respectively.

### 3.1. Subjective Outcomes

**Figure 3:**
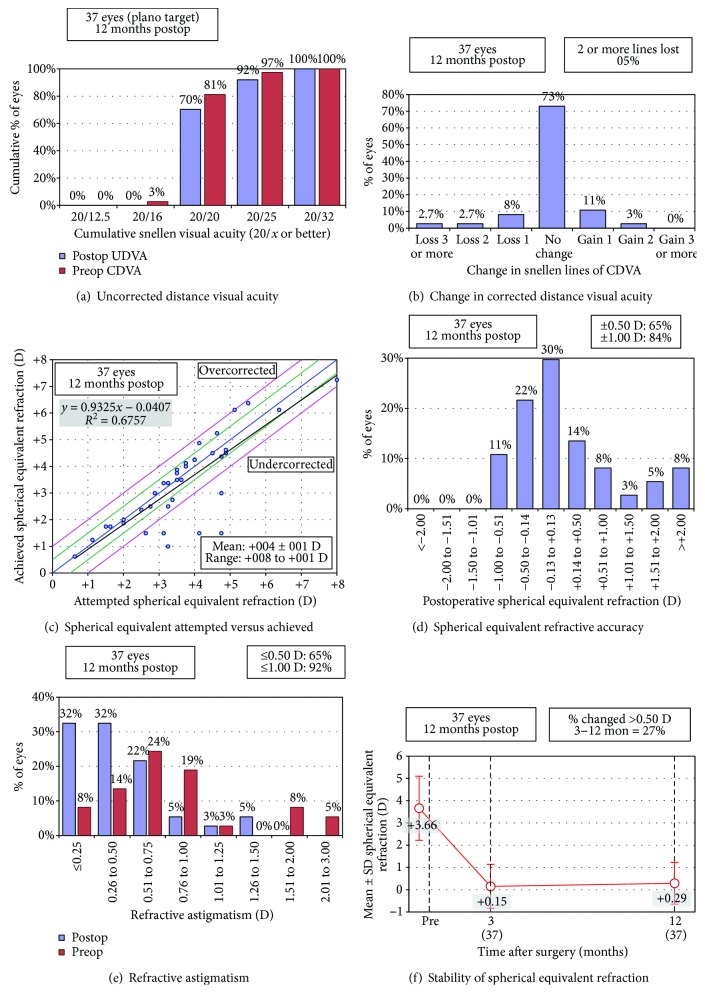
Clinical outcomes. Postoperative follow-up results (12 months after surgery). (a) Postoperative uncorrected visual acuity compared with the preoperative corrected distance visual acuity (CDVA). (b) Changes in lines of CDVA. (c) Intended versus achieved correction (manifest refraction). (d) Distribution of postoperative spherical equivalent (predictability). (e) Distribution of preoperative and postoperative astigmatism. (f) Stability of the manifest refraction.

Outcomes including the preop moment are summarized in Table 1, and Figure 3 in terms of efficacy (Figure 3(a)), safety (Figure 3(b)), predictability (Figure 3(d)), and astigmatism (Figure 3(e)), between the pre- and postop moments, showed a statistically significant change (improvement in visual acuity and decrease in astigmatism and ametropia).

As per the change in the subjective refraction SE over the follow-up period after surgery (Figure 3(f)), a 0.14 D of regression was detected which was not statistically significant.

Subjective outcomes in subgroups 1 and 2 are summarized in (Tables 2 and 3), respectively.

### 3.2. Objective Outcomes

**Figure 4:**
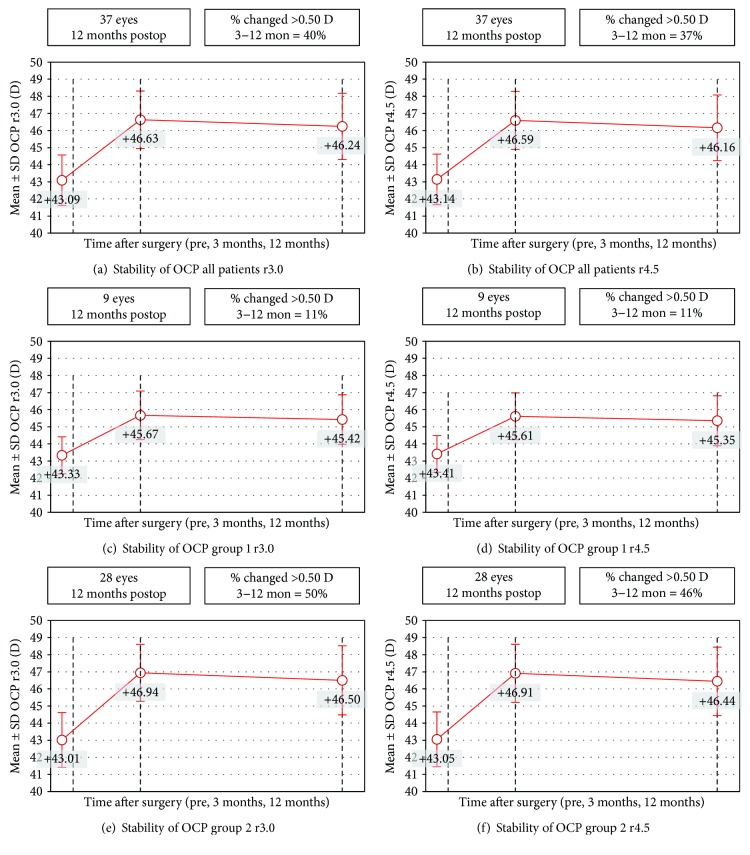
OCP stability.

Objective outcomes are also summarized in (Table 1) and in (Figure 4). The objective corneal power (OCP) calculated objectively by the ray tracing from the corneal topographies showed a statistically significant change (shift) in the corneal power comparing the pre- and the two postop moments analyzed. And, there was no statistically significant difference between the postsurgery moments analyzed.

In all patient groups, OCP had 0.39 D and 0.41 D of regression considering a 6 mm and 9 mm central corneal zone (Figures 4(a) and 4(b)). In group 1, OCP regression was 0.25 D and 0.26 D with 6 and 9 mm central corneal zone, respectively (Figures 4(c) and 4(d)). And in group 2, OCP regression was 0.44 D and 0.47 D with 6 mm and 9 mm central corneal zone (Figures 4(e) and 4(f)). All these regression values are by considering the postop follow-up period (12 month). The pairwise relationship between the preop moment and the other two is statistically significant. And none of the *p* values related to pairwise relationship between the postop moments are under the cutoff value (0.05 or 0.017 with Bonferroni correction).

## 4. Discussion

Laser in situ keratomileusis (LASIK) ablation to correct hyperopia and hyperopic astigmatism has reported acceptable efficacy for corrections up to 4.0 diopters (D) [1, 26, 28]. Some studies reported lower predictability, safety, and refractive stability for higher corrections [3, 5]. Other studies though claim that hyperopic treatment by LASIK is not stable overtime [23, 29]. As per the US Food and Drug Administration's (FDA) definition, stability is described as change in the patient's manifest refraction which is <1.0 D over 12 months' period.

Regression is still considered one of the most important challenges post-LASIK ablation for hyperopia. Most of the regression was reported to occur within the first 12 months posttreatment [3, 6, 7, 30]. Recently, Dave et al. [26] reported an increase in hyperopia of +1.47 D ± 1.43 between 1 year and 16 years (*p* < 0.0001) and of +1.13 ± 0.8 D between 5 years and 16 years (*p* < 0.03) Waring et al. [30] reported that most of the hyperopic shift experienced postoperatively in his cohort occurred during the first 3 months with a mean change in SE of 0.11 D occurring between the first and third month postoperatively. Plaza-Puche et al. [7] reported that refractive stability showed a significant regression in the first 12 months after surgery (*p* < 0.01) with more stable results from 12 months postoperatively (*p* = 0.08). de Ortueta and Arba Mosquera [6] and Waring et al. [30] showed similar results within 3 months postoperatively, the intended corneal curvature is achieved, and only small changes occur after this time. Plaza-Puche et al. [7] reported regression of 0.47 D between 3 and 36 months' post LASIK treatment for hypermetropia. de Ortueta and Arba Mosquera [6] reported a regression of 0.006 D/month between 3 and 12 months postoperatively. Less regression could be observed with the use of larger ablation zones [29] as reported also by Zaldivar et al. [31]. These results correspond with our results. In our study, the UDVA (logMAR) improved from 0.29 ± 0.31 preoperatively to 0.06 ± 0.08 at the end of the follow-up period. This is considered as one of the potential advantages of the 6th generation Excimer when used to treat hyperopia.

In light propagation analysis, we calculate the diopter power of the cornea in isolation of other ocular structures, so any change in this D power during the follow-up period is exclusively due to corneal changes. In our study this change was not clinically significant for all the patients *p* = 0.09, 0.06 for the 6 mm, 9 mm central corneal zones, respectively (Significance was set with Bonferroni correction *p* < 0.017). Studying this change over a 9 mm zone is needed in these patients as hyperopic LASIK correction is done as a peripheral annular zone.

Even when subdividing our patients into subgroups according to their SE, the changes for the 6 mm as well as the 9 mm central corneal zone were not statically significant. In our OCP results, we noticed a higher regression when the corneal area studied is larger. None of the OCP values were beyond the 1.0 D/year permitted by the FDA.

These results suggest that regression is positively correlated with the amount of the SE intended to correct. When stability is studied by subjective refraction, we can see in our subjective results a lower quantity of regression compared with the objective data. This could be caused by small amounts of accommodation or a higher amount of spherical aberration on LASIK-corrected corneas; this may introduce a bias that minimizes the real change on the dioptric power of the cornea. Stability could be studied objectively by topographic *K* values (means), but “means” could not detect central-peripheral compensated changes in corneal curvature. Therefore, a deeper study on the behavior of light passing through an ablated cornea could help to understand the regression patterns.

A variety of theories are available to explain regression post-LASIK ablation for hyperopia. This might be related to wound modeling. Initially, excimer laser ablation induces keratocyte apoptosis [32]; this will be followed by myofibroblasts proliferation causing stromal remodeling. A second theory relates this regression to epithelial thickness changes. As paracentral epithelium thickens to compensate for the LASIK effect [26], this will reduce some of the shape changes caused by stromal ablation due to the creation of thinning in the central cornea and flattening of it consequently. A third mechanism might be related to expansion of the midperipheral corneal fibers which have been ablated by laser. As these fibers expand, this will consequently lead to flattening of the steepened central cornea. However, as the keratometry did not appear to change over the 12-month period, this suggests that all these theories fail to explain the exact cause of regression post-LASIK for hypermetropia. The most accepted mechanism for this regression might be related to the cilio-zonular-lenticular effect. A very reasonable explanation for early regression post-LASIK ablation for hypermetropia could be inadequate treatment of latent hyperopia.

This hyperopic change should of course be differentiated from late regression which occurs years after LASIK ablation for hypermetropia. This might be related to the physiologic hyperopic shift that is expected to occur with time. Attebo et al. [33] found that hyperopia prevalence and age had a statistically significant relation (*p* < 0.05), increasing from 36% in patients aged < 60 years to 71% in subjects aged ≥ 80. Lee et al. [34] and Guzowski et al. [35] observed a small hyperopic shift of 0.12 D and 0.19 D, respectively, across the entire population of patients over a 5-year period. Another explanation for this late regression might be inadequate treatment of latent hyperopia that became manifest as accommodation declines with aging [28].

Alio et al. followed their hyperopic patients for 16 years post-LASIK correction [28]. They found a significant increase in SE at end of follow-up in comparison to 1 year posttreatment. This effective loss of the procedure is consistent with the normal physiologic changes expected with age after the first 5 years. Applying the principle of ray tracing to track the possible changes that might occur on the treated cornea overtime will help us understand more the probable behavior of the cornea post-LASIK treatment. A larger sample size with longer follow-up period is very helpful to study corneal stability following hyperopic LASIK treatment using this method of analysis and to prove its effectiveness.

A common problem encountered by patients post-LASIK surgery is that either they are happy patients and do not come back for follow-ups or they are not totally happy and then seek help in other locations. This might explain the small sample size (only 37 patients). Even patients who had their laser refractive surgery performed by residents showed high patient satisfaction and an improved RQL 1 year postoperatively [36]. Except the recently published study by Dave et al. [26], few studies reported more than 5 years and none more than 10-year follow-up post-LASIK hyperopic refraction. As studies support that most of the regression occurs during the first 12 months, we thought this might be a good start to apply ray tracing methodology in calculating (through a purely objective way) the contribution of corneal changes in posthyperopic LASIK correction regression.

In summary, the use of light propagation analysis of the anterior corneal surface, as used in this report, to test stability of the anterior corneal surface profile following 6th-generation LASIK for hyperopic correction shows stable anterior corneal surface over a 12-month period following LASIK hyperopic correction. This finding is relevant as it indicates a good efficiency of recently developed excimer laser technology in the correction of hyperopia with LASIK.

## Figures and Tables

**Table 1:** Preoperative and postoperative visual, refractive, and objective outcomes 12 months after hyperopic LASIK^a,b,c^.

	Pre	3 months	12 months	*p* ^b^ (pre/3 mon/12 mon)	*p* ^c^ (pre/3 mon)	*p* ^c^ (pre/12 mon)	*p* ^c^ (3 mon/12 mon)
Patients	37						
Age	35.35 ± 9.23 (18 to 51)						
UDVA (logMAR)	0.29 ± 0.31 (−0.08 to 1.30)	0.06 ± 0.08 (0.00 to 0.40)	0.03 ± 0.06 (0.00 to 0.22)	<0.01	<0.01	<0.01	1.0
CDVA (logMAR)	0.02 ± 0.03 (−0.08 to 0.15)	0.02 ± 0.04 (0.00 to 0.15)	0.02 ± 0.05 (0.00 to 0.22)	0.50	1.0	0.85	1.0
SPH (D)	4.46 ± 1.47 (1.75 to 8.50)	0.45 ± 0.99 (−1.25 to 3.50)	0.55 ± 0.93 (−1.00 to 3.00)	<0.01	<0.01	<0.01	1.0
CYL (D)	−1.57 ± 1.27 (−4.75 to 0.00)	−0.60 ± 0.42 (−2.00 to 0.00)	−0.55 ± 0.35 (−1.50 to 0.00)	<0.01	0.01	<0.01	1.0
SE (D)	3.66 ± 1.44 (0.63 to 8.00)	0.15 ± 0.99 (−1.38 to 3.00)	0.29 ± 0.94 (−1.00 to 3.25)	<0.01	<0.01	<0.01	0.83
OCP r3 (D)	43.09 ± 1.48 (40.64 to 47.91)	46.63 ± 1.68 (43.63 to 50.91)	46.24 ± 1.94 (41.61 to 50.68)	<0.01	<0.01	<0.01	0.09
OCP r4.5 (D)	43.14 ± 1.48 (40.74 to 48.01)	46.59 ± 1.70 (43.46 to 50.90)	46.16 ± 1.92 (41.60 to 50.68)	<0.01	<0.01	<0.01	0.06

UDVA = uncorrected distance visual acuity; CDVA = corrected distance visual acuity; SPH = sphere; CYL = cylinder; SE = spherical equivalent; OCP r3 = objective corneal power radius 3 mm; OCP r4.5 = objective corneal power radius 4.5 mm. ^a^Values shown as mean ± standard deviation (range). ^b^ANOVA for paired data. ^c^Post hoc with Bonferroni correction.

**Table 2:** Preoperative and postoperative visual, refractive, and objective outcomes 12 months after hyperopic LASIK^a,b,c^ (group 1, SE ≤ 3).

	Pre	3 months	12 months	*p* ^b^ pre/3 mon/12 mon)	*p* ^c^ (pre/3 mon)	*p* ^c^ (pre/12 mon)	*p* ^c^ (3 mon/12 mon)
Eyes (*n*)	9						
Age	37.56 ± 7.41 (27 to 45)						
UDVA (logMAR)	0.29 ± 0.28 (0.00 to 0.82)	0.04 ± 0.05 (0.00 to 0.15)	0.03 ± 0.07 (0.00 to 0.22)	<0.01	<0.01	<0.01	0.40
CDVA (logMAR)	0.01 ± 0.02 (0.00 to 0.07)	0.01 ± 0.02 (0.00 to 0.05)	0.02 ± 0.07 (0.00 to 0.22)	1.0	1.0	1.0	1.0
SPH (D)	3.03 ± 0.88 (1.75 to 4.50)	0.25 ± 0.31 (−0.25 to 0.75)	0.33 ± 0.48 (0.00 to 1.50)	<0.01	<0.01	<0.01	0.69
CYL (D)	−2.31 ± 1.45 (−4.75 to −0.50)	−0.53 ± 0.29 (−1.00 to −0.25)	−0.50 ± 0.27 (−1.00 to −0.25)	<0.01	0.02	0.01	1.0
SE (D)	1.88 ± 0.73 (0.63 to 2.88)	0.01 ± 0.43 (−0.50 to 0.63)	0.08 ± 0.40 (−0.25 to 1.13)	<0.01	<0.01	<0.01	0.53
OCP r3 (D)	43.33 ± 1.08 (41.91 to 44.67)	45.67 ± 1.42 (43.63 to 47.52)	45.42 ± 1.45 (43.21 to 47.63)	<0.01	<0.01	<0.01	0.30
OCP r4.5 (D)	43.41 ± 1.07 (42.01 to 44.76)	45.61 ± 1.37 (43.53 to 47.57)	45.35 ± 1.46 (43.01 to 47.52)	<0.01	<0.01	<0.01	0.36

UDVA = uncorrected distance visual acuity; CDVA = corrected distance visual acuity; SPH = sphere; CYL = cylinder; SE = spherical equivalent; OCP r3 = objective corneal power radius 3 mm; OCP r4.5 = objective corneal power radius 4.5 mm. ^a^Values shown as mean ± standard deviation (range). ^b^Friedman test. ^c^Wilcoxon test with Bonferroni correction (*p* < 0.017).

**Table 3:** Preoperative and postoperative visual, refractive and objective outcomes 12 months after hyperopic LASIK^a,b,c^ (group 2, SE > 3).

	Pre	3 months	12 months	*p* ^b^ (pre/3 mon/12 mon)	*p* ^c^ (pre/3 mon)	*p* ^c^ (pre/12 mon)	*p* ^c^ (3 mon/12 mon)
Eyes (*n*)	28						
Age	34.64 ± 9.75 (18 to 51)						
UDVA (logMAR)	0.30 ± 0.33 (−0.08 to 1.30)	0.06 ± 0.09 (0.00 to 0.40)	0.03 ± 0.05 (0.00 to 0.15)	<0.01	<0.01	<0.01	0.31
CDVA (logMAR)	0.01 ± 0.04 (−0.08 to 0.15)	0.02 ± 0.05 (0.00 to 0.15)	0.02 ± 0.04 (0.00 to 0.14)	0.66	1.0	1.0	1.0
SPH (D)	4.88 ± 1.35 (3.50 to 8.50)	0.51 ± 1.13 (−1.25 to 3.50)	0.62 ± 1.03 (−1.00 to 3.00)	<0.01	<0.01	<0.01	1.0
CYL (D)	−1.33 ± 1.13 (−4.25 to 0.00)	−0.61 ± 0.41 (−2.00 to 0.00)	−0.57 ± 0.38 (−1.50 to 0.00)	<0.01	0.02	<0.01	1.0
SE (D)	4.22 ± 1.12 (2.75 to 8.00)	0.21 ± 1.13 (−1.38 to 3.00)	0.35 ± 1.06 (−1.00 to 3.25)	<0.01	<0.01	<0.01	1.0
OCP r3 (D)	43.01 ± 1.60 (40.64 to 47.91)	46.94 ± 1.66 (44.46 to 50.91)	46.50 ± 2.02 (41.61 to 50.68)	<0.01	<0.01	<0.01	0.17
OCP r4.5 (D)	43.05 ± 1.60 (40.74 to 48.01)	46.91 ± 1.70 (44.27 to 50.90)	46.44 ± 2.00 (41.60 to 50.68)	<0.01	<0.01	<0.01	0.13

UDVA = uncorrected distance visual acuity; CDVA = corrected distance visual acuity; SPH = sphere; CYL = cylinder; SE = spherical equivalent; OCP r3 = objective corneal power radius 3 mm; OCP r4.5 = objective corneal power radius 4.5 mm. ^a^Values shown as mean ± standard deviation (range). ^b^ANOVA for paired data. ^c^Post hoc with Bonferroni correction.
